# The complete chloroplast genome of the marsh plant-*Leucanthemella linearis* (Asteraceae)

**DOI:** 10.1080/23802359.2018.1437801

**Published:** 2018-02-13

**Authors:** YuePing Ma, ShengYi Sun, Liang Zhao

**Affiliations:** a College of Life and Health Sciences, Northeastern University, Shenyang, China;; b College of Life Sciences, Northwest A&F University, Yangling, China

**Keywords:** *Leucanthemella linearis*, chloroplast genome, marsh plant

## Abstract

*Leucanthemella linearis* is an important marsh plant. The complete chloroplast genome sequence of *L*. *linearis* was obtained using next generation sequencing. It was 15,1401 bp in length, including a pair of inverted repeat (IR, 24,941 bp) regions separated by a small single copy (SSC, 18,392 bp) sequence and a large single copy (LSC, 83,127 bp) sequence. The cp genome contained 140 genes, consisting of 96 protein-coding genes, 8 rRNA genes and 36 tRNA genes. Twenty-two genes were present in the IR region. Thirty-four SSR sites were detected in the cp genome. Phylogenetic analysis with the reported chloroplast genomes revealed that *L*. *linearis* is most closely related to Tribe Heliantheae species. This new data will help to understand the phylogenetic position and biology of the *Leucanthemella*.

The growth of the population and the acceleration of industrialization destroyed the ecological environment. The wetland areas have shrunk gradually and the biodiversity of wetland is decreasing and some species are even extinct which thus threatened our sustainability (Niu et al. 2012; Zhou et al. 2014).

*Leucanthemella linearis* (Matsum.) Tzvel. (2*n* = 18) is distributed in East Asia from Manchuria, China, Korea, to Japan and grows in marsh areas. As perennial plants, *L. linearis* has the pretty scene value for these wetlands. Here we obtained the complete chloroplast genome of *L. linearis* using the Illumina HiSeq platform. The cp genome sequence of *L*. *linearis* will not only protect the genetic resource of wetland, but also help to clarify phylogenetic relationships of the Tribe Anthemideae.

Fresh leaves of *L. linearis* were collected from Hani wetland (Jilin, China) and the voucher specimen was deposited into Herbarium of Northwest A&F University (China). Total genomic DNA was extracted from fresh leaves using CTAB method (Doyle and Doyle 1987) with some modification.

Then the DNA was sent to GENEWIZInc (Jiangsu, China) for library construction and sequencing. Paired-end reads of 2 × 300 were generated on an Illumina MiSeq Sequencer. Approximately 3.0 Gb of clean reads data were generated for *L. linearis* after trimming with Trimmomatic v0.36 (http://www.sadellab.org/cms/index.php?page=trimmomatic) (Bolger et al. 2014). *De novo* and reference-guided methods were combined to assemble chloroplast genome using Geneious10.1.3 (Kearse et al. 2012). The complete chloroplast genomes were annotated using the webserver DOGMA (Wyman et al. 2004) and the sequence was deposited in GenBank with the accession number MG748695. Simple sequence repeat (SSR) was investigated using Gramene (http://www.gramene.org/db/markers/ssrtool) (Temnykh et al. 2001). The complete cp genome of *L*. *linearis* was 151,401 bp in length, including a pair of inverted repeat (IR, 24,941 bp) regions seperated by a small single copy (SSC, 18,392 bp) sequence and a large single copy (LSC, 83,127bp) sequence (Figure 1). The GC content of cp genome in the *L*. *linearis* is 37.3% and the corresponding values in LSC, SSC and IR regions are 35.4, 30.7 and 43%, respectively. The complete cp genome contained 140 genes, including of 96 protein-coding genes, 8 rRNA genes and 36 tRNA genes. Twenty-two genes were present in the IR region. Thirty-four SSR sites were detected in the cp genome of *L*. *linearis*. Most of them were composed of A or T base and dimer and tetramer were the majority.

**Figure 1.**
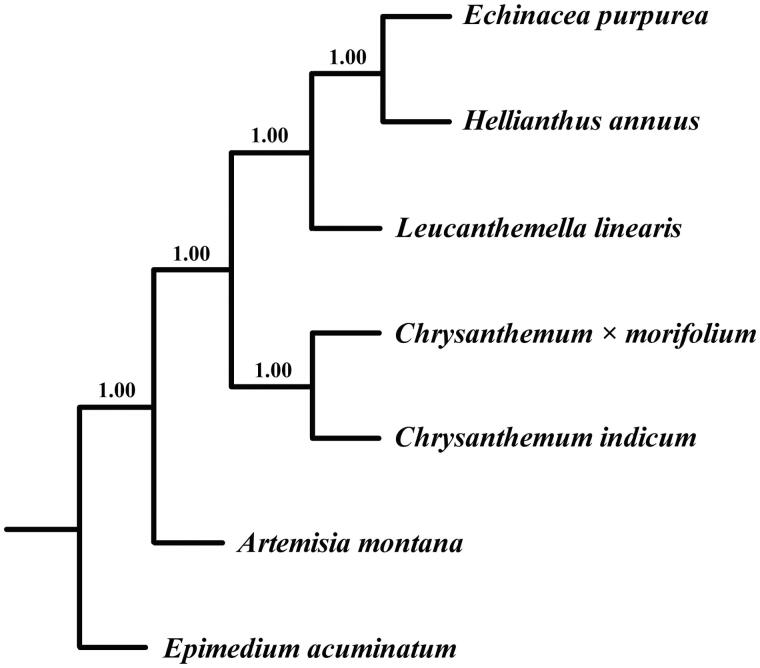
Bayesian 50% majority-rule consensus tree of *L*. *linearis* with 5 Asteraceae species inferred from whole chloroplast genomes. Bayesian posterior probabilities are shown above the branches.

The phylogenetic relationship between *L*. *linearis* and related species was performed with MrBayes3.2.1 (Ronquist et al. 2012) using six chloroplast genomes of Asteraceae aligned with MAFFT (Katoh and Toh 2010). *Epimedium acuminatum* was added as the outgroup. The phylogenetic analysis reveals that *L*. *linearis* was closely related to *Helianthus* tribe species (Figure 1). The chloroplast resource will provide molecular genetic information for DNA barcoding, conservation genetics, and breeding of *L*. *linearis* in the future.
